# First record of *Xestochironomus* Sublette and Wirth, 1972 (Chironomidae: Chironominae) in the Mexican Nearctic with notes on their habitat

**DOI:** 10.3897/BDJ.7.e32124

**Published:** 2019-04-01

**Authors:** Orestes Carlos Bello-González, Perla Alonso-EguíaLis, Norman Mercado-Silva

**Affiliations:** 1 Maestría en Biología Integrativa de la Biodiversidad y la Conservación. Centro de Investigación en Biodiversidad y Conservación (CIByC). Universidad Autónoma del Estado de Morelos. Av. Universidad 1001, Col. Chamilpa, C.P. 62209, Cuernavaca, Morelos, Mexico Maestría en Biología Integrativa de la Biodiversidad y la Conservación. Centro de Investigación en Biodiversidad y Conservación (CIByC). Universidad Autónoma del Estado de Morelos. Av. Universidad 1001, Col. Chamilpa, C.P. 62209 Cuernavaca, Morelos Mexico; 2 Laboratorio de Bioindicadores. Instituto Mexicano de Tecnología del Agua. Paseo Cuauhnáhuac 8532. C.P 62550. Progreso, Jiutepec, Morelos, Mexico Laboratorio de Bioindicadores. Instituto Mexicano de Tecnología del Agua. Paseo Cuauhnáhuac 8532. C.P 62550. Progreso Jiutepec, Morelos Mexico; 3 Centro de Investigación en Biodiversidad y Conservación (CIByC). Universidad Autónoma del Estado de Morelos. Av. Universidad 1001, Col. Chamilpa, C.P. 62209, Cuernavaca, Morelos, Mexico Centro de Investigación en Biodiversidad y Conservación (CIByC). Universidad Autónoma del Estado de Morelos. Av. Universidad 1001, Col. Chamilpa, C.P. 62209 Cuernavaca, Morelos Mexico

## Abstract

We report the first record of *Xestochironomus* Sublette and Wirth, 1972 for the Mexican Nearctic. Larvae of *Xestochironomus* are known from the Neotropics and Nearctic regions. We report them for the Sonora river, NW Mexico, 300 km SW from the closest previous record in the U.S. Habitat data are provided and discussed. Our finding provides supporting evidence for the continuous presence of the genus throughout the Americas, including desert systems.

## Introduction

The Chironomidae are widely distributed throughout the world (Ashe et al. 1987, Ferrington 2008). Chironomids are one of the most diverse and ecologically important groups found in freshwater systems. They play key roles in community and ecosystem functioning (Porinchu and MacDonald 2003).

*Xestochironomus* Sublette and Wirth, 1972 substituted *Insulanus* Sublette, 1967 after a study on adults mostly from the Antillean islands (Sublette and Wirth 1972). The genus includes species with larvae highly specialized on xylophagy, inhabiting lotic environments with little human disruption in the American continent (Borkent 1984). *Xestochironomus* has mainly been reported for tropical environments in the Neotropicsand in the S and SW United States. (Nearctic region) (Borkent 1984, Hudson et al. 1990, Pinho and Souza 2013). With Mexico being a transition zone between the neotropic and nearctic zoogeographic regions, presence of a member of the genus was expected. It was, however, not reported in Reiss (1982), Spies and Reiss (1996) or Spies et al. (2009), the main sources for data on Chironomidae from Mexico. The genus has only been reported once in Mexico, for the Calakmul Biosphere Reserve in the southern State of Campeche (Contreras-Ramos and Andersen 1999), >2000 km SE from our current record. This is thus the first record for *Xestochironomus* for the Mexican Nearctic.

## Methods

**Figure 1.**
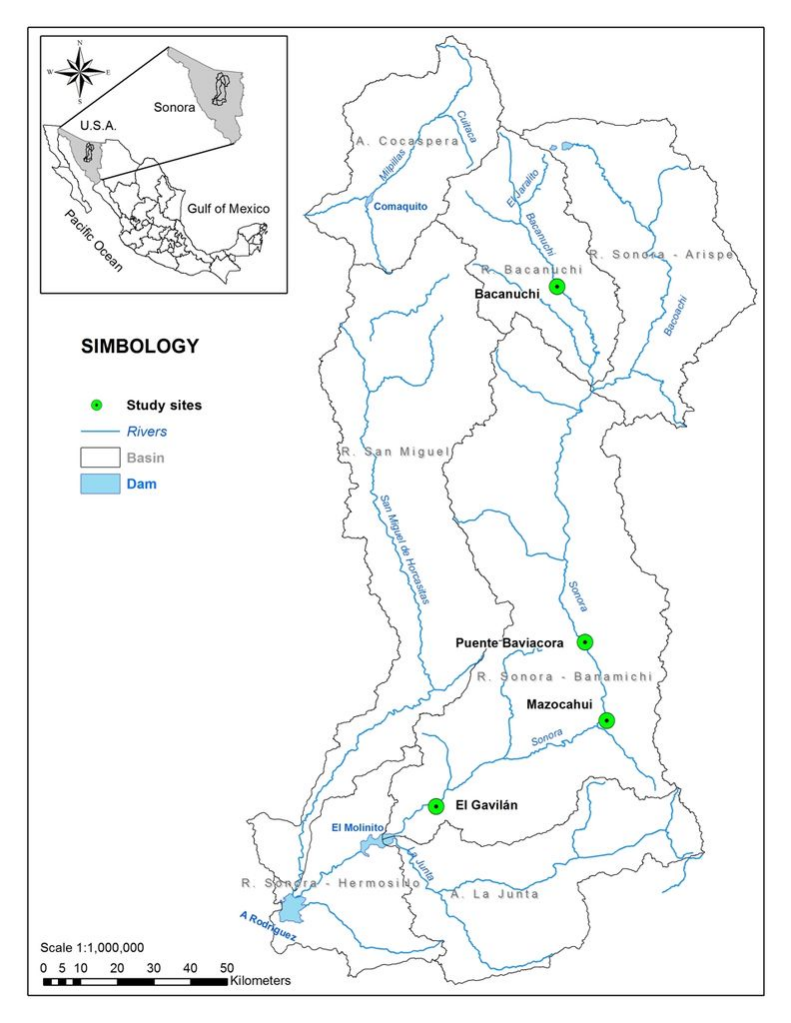
Localities where *Xestochironomus* larvae were captured in the Sonora River, north-western Mexico.

Collection sites are located in the Sonora and Bacanuchi rivers (Fig. 1). Samples were obtained in November 2017 with a D-net (40 cm wide; 0.5 mm mesh) and via vigorous washing of woody debris hand-picked from the collection site. Samples were preserved in 80% ethanol. In the laboratory, individuals were separated from the debris and mounted on microscope slides in Euparal following Saether (1969). Slides were then examined under an optical microscope (Zeiss, model: Primo Star) with Nomarsky phase contrast and 1000x magnification with an immersion oil objective, coupled to a AxioCam ERc 5s camera. Borkent (1984), Epler (2001) and Ferrington et al. (2008) were used for specimen identification.

Habitat variables were obtained during the field collections. Bottom substrate was classified using Wentworth’s scale (Cummins 1996). Temperature (°C), pH, dissolved oxygen (mg/l) and conductivity (units) were obtained with a YSI Professional Plus (Xylem Inc) multimeter. Water depth and velocity at the collection site were measured with a Flowmate 2000 (Marsh-McBirney Inc) flowmeter.

## *Xestochironomus* sp.

### Materials examined

Mexico: Sonora: Bacanuchi (30°35'57"N, -110°14'36"W), collectors: P. Alonso-EguíaLis and O. Bello, 9 individuals. Deposited at the scientific collection of the Laboratorio de Bioindicadores of the Instituto Mexicano de Tecnología del Agua. Captured 18 November 2017.

México: Sonora: Puente Baviacora (29°43'32"N, -110°10'30"W), collectors: P. Alonso-EguíaLis and O. Bello, 3 individuals. Deposited at the scientific collection of the Laboratorio de Bioindicadores of the Instituto Mexicano de Tecnología del Agua. Captured 12 November 2017.

México: Sonora: Mazocahuí (29°31'58"N, -110°07'17"W), collectors: P. Alonso-EguíaLis and O. Bello, 1 individual. Deposited at the scientific collection of the Laboratorio de Bioindicadores of the Instituto Mexicano de Tecnología del Agua. Captured 12 November 2017.

México: Sonora: El Gavilán (29°19'18"N, -110°32'25"W), collectors: P. Alonso-EguíaLis and O. Bello, 1 individual. Deposited at the scientific collection of the Laboratorio de Bioindicadores of the Instituto Mexicano de Tecnología del Agua. Captured 17 November 2017.

### Identification

*Xestochironomus* larvae are similar to those of *Stenochironomus* Kieffer, 1919. However, they can be distinguished by the following attributes (all of which were present in the individuals used for this article): sclerotised mentum concave with well sclerotised teeth (Fig. 2a); mentoventral plates vestigial; anal tubules elongated with 4-5 constrictions (Fig. 2b); cephalic capsule dorsoventrally flattened with a Y-shaped dorsal design (Fig. 2c) and antennal blade extending beyond the apex of the third antennal segment (Fig. 2d). In contrast, *Stenochironomus* larvae have 10-12 teeth in the mentum, the antennal blade reaches only the apex of the 2nd antennal segment and the anal tubules have, at most, two constrictions.

### Environmental variables

Habitat variables for collection sites are presented in Table 1.

## Discussion

Members of *Xestochironomus* are known from the Americas with most records from neotropical areas in Brazil, Chile, Colombia, Costa Rica, Cuba, Dominica, Guatemala, Jamaica, Panama, Peru, Puerto Rico and Venezuela. Nearctic records are all from the USA in Florida, South Carolina, Georgia, Texas and New México (Andersen and Kristoffersen 1998, Bello-González et al. 2016, Borkent 1984, Hudson et al. 1990, Pinho and Souza 2013, Ruiz-Moreno et al. 2000, Sublette and Sasa 1994, Sublette and Wirth 1972). The nearest record to the one presented here is from approximately 300 km away in the San Francisco Hot Springs area of the Gila River in New Mexico (Borkent 1984). In Mexico, the only record is for the Calakmul biosphere reserve in the Yucatan Peninsula (Contreras-Ramos and Andersen 1999), over 2000 km SE of our record.

Following Abell et al. (2008) ecoregion classification, most *Xestochironomus* records are from humid tropical or subtropical areas. Some of the species in this genus can be very abundant in tropical streams draining rainforests (Ferrington et al. 1993, Grund 2006). These conditions favour woody debris inputs to the channel, which constitute both habitat and a food resource for larvae of *Xestochironomus* (Epler 2001, Sanseverino and Nessimian 2008). High densities in larval xylophagous chironomids are found under such conditions (Cranston 2008).

The Sonora River basin, located in the Gulf of California climatic province, has a distinctive dry climate (Vidal 2005). Vegetation types dominating this area include shrubs and herbs (Martínez-Yrízar et al. 2009) with Cottonwood (*Populus* sp.) being an important component of the riparian vegetation. These dry conditions lead to relatively low woody debris inputs to stream channels (Bunn et al. 2006, Davies et al. 1995, Jones 1997, Cushing and Allan 2001). Our record from the Sonora and records from the Gila system nevertheless confirm that larvae of *Xestochironomus* can occur in streams located in arid regions with little input of woody debris. It is thus possible that the genus has a continuous distribution from the Neotropics to the Nearctic region, with deserts in N Mexico and SW USA not being barriers to their distribution.

Most (12/14) captured larvae were collected from sites Bacanuchi and Puente Baviacora, while only one individual was captured in sites Mazocahui and El Gavilan despite all sites having similar sampling efforts. Other than the information related to trophic habits, very little data exists about the overall conditions of the habitat required by *Xestochironomus* larvae. Since they typically inhabit galleries in submerged wood, their low mobility could render them sensitive to changes in local conditions. Xylophagous larvae usually require good water quality (Borkent 1984, Cranston 2008). Mazocahui and El Gavilan had relatively high conductivity (>1000 μS/cm), values above that threshold are typically associated with pollution (Chapman and Kimstach 1996). Low dissolved oxygen and high temperatures in these sites might also have resulted in little oxygen being available for aquatic fauna. Thus, lower water quality in Mazocahui and El Gavilan might be a cause for *Xestochironomus* having a relatively lower abundance in these sites.

## Figures and Tables

**Figure 2a.**
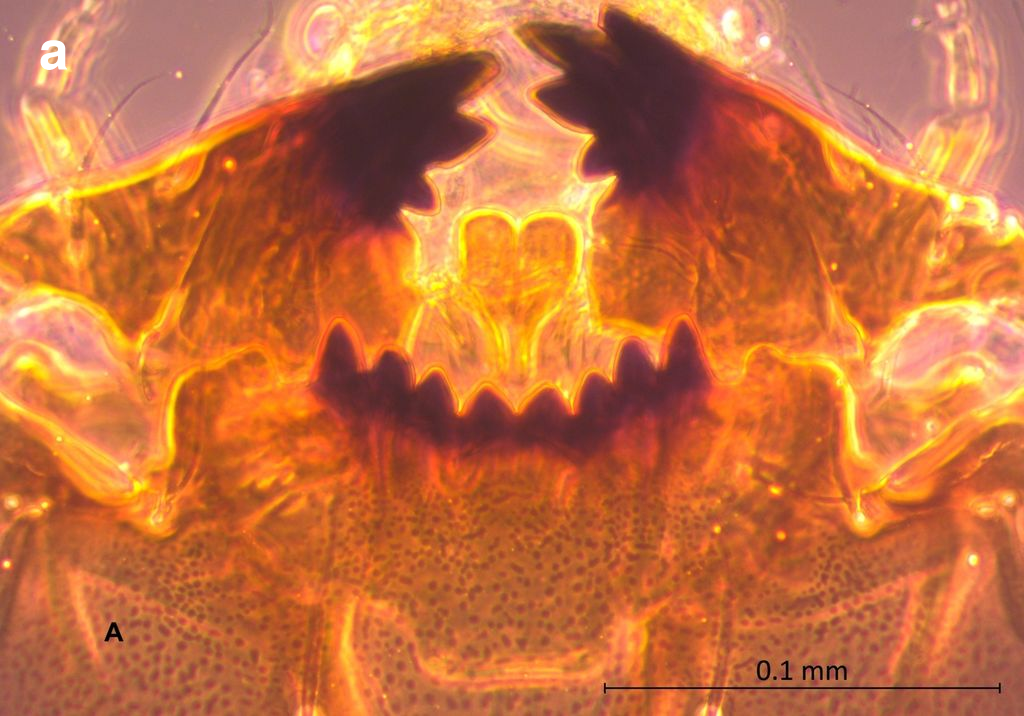
Mouth parts

**Figure 2b.**
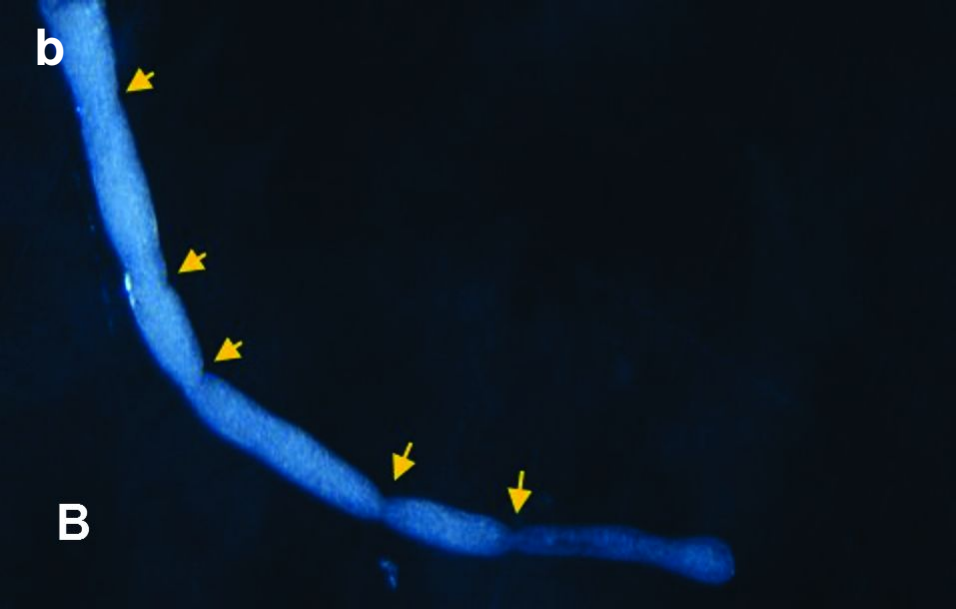
Anal tubule showing constrictions

**Figure 2c.**
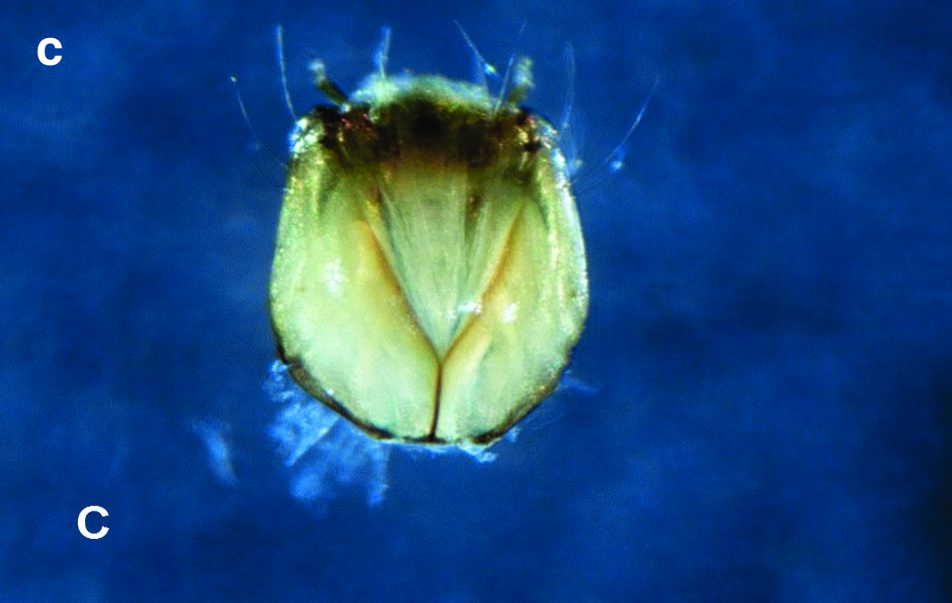
Dorsal view of head showing Y-shaped suture

**Figure 2d.**
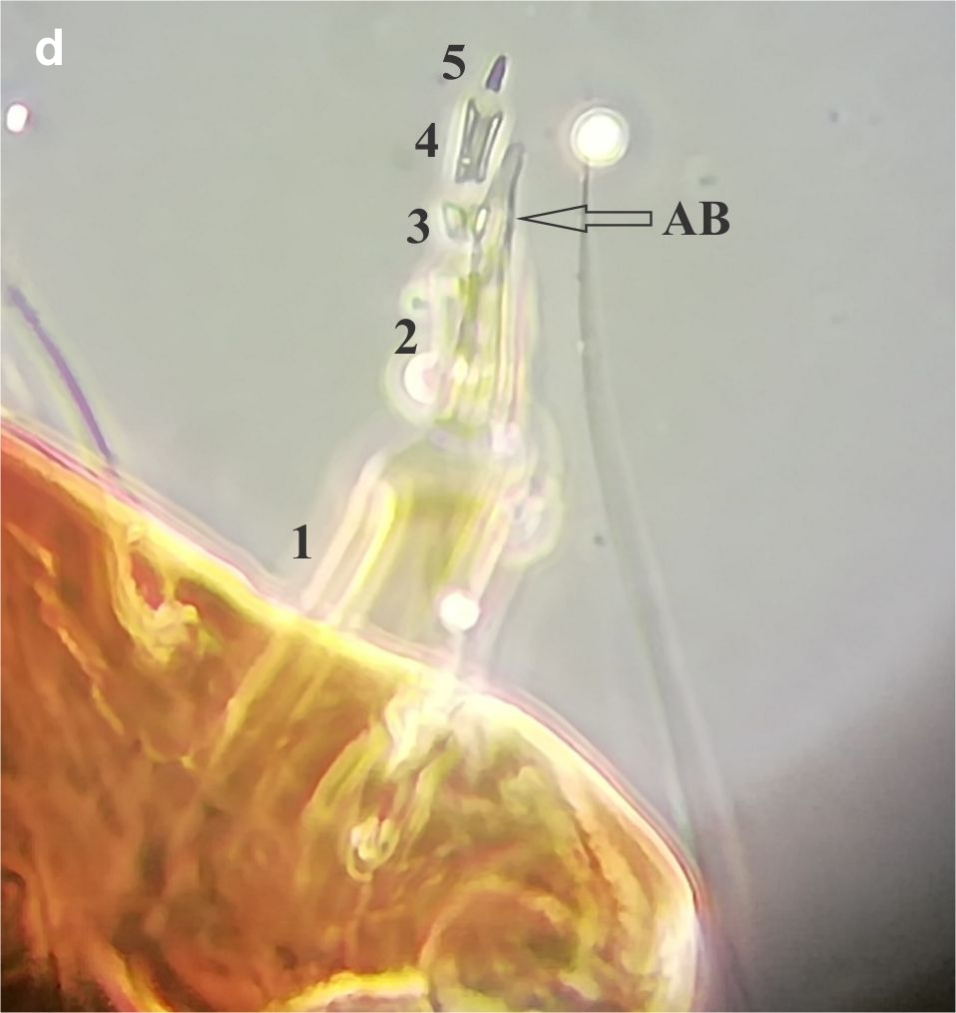
Antenna. 1-5: antennal segments, AB: antennal blade

**Table 1.** Habitat data for sites where *Xestochironomus* larvae were found. Site = SIS; Bacanuchi = BC; Puente Baviacora = PB; Mazocahui = MZ; El Gavilán = EG; Conductivity = Cond; Dissolved oxygen = DO; temperature = T; depth = D; water velocity = V; substrate = S; sand = SN; gravel = GV.

**SIS**	**Altitude (m)**	**Cond (μS/cm)**	**pH**	**DO (mg/l)**	**T (° C)**	**D (cm)**	**V (m/s)**	**S**
BC	1030	784	7.4	6.22	23.4	10	0.15	SN-GV
PB	552	860	7.1	4.26	24.2	8	0.44	SN
MZ	473	1249	7.9	3.48	30.9	5	0.20	SN
EG	328	1508	7.8	3.23	27.2	9	0.16	SN
